# Identification of *Pentatrichomonas hominis* in preputial washes of bulls in Brazil

**DOI:** 10.1590/S1984-29612022034

**Published:** 2022-07-04

**Authors:** Otávia Reis e Silva, Laura Ribeiro, Vera Lucia Teixeira de Jesus, Douglas McIntosh, Lara Nogueira Silenciato, Joaquim Esquerdo Ferreira, Marco Roberto Bourg de Mello

**Affiliations:** 1 Laboratório de Doenças da Reprodução, Instituto de Veterinária, Universidade Federal Rural do Rio de Janeiro – UFRRJ, Seropédica, RJ, Brasil; 2 Laboratório Multiusuário de Biologia Molecular, Departamento de Parasitologia Animal – DPA, Instituto de Veterinária, Universidade Federal Rural do Rio de Janeiro – UFRRJ, Seropédica, RJ, Brasil; 3 Departamento de Reprodução e Avaliação Animal – DRAA, Instituto de Zootecnia – IZ, Universidade Federal Rural do Rio de Janeiro – UFRRJ, Seropédica, RJ, Brasil; 4 Faculdade de Medicina Veterinária, Centro Universitário de Valença – UNIFAA, Valença, RJ, Brasil

**Keywords:** Nelore bulls, Trichomonads, Pentatrichomonas hominis, PCR, Touros Nelore, Trichomonadida, Pentatrichomonas hominis, PCR

## Abstract

The parabasalid *Pentatrichomonas hominis* is generally considered to represent a symbiotic component of the gastrointestinal microbiota in a wide variety of vertebrate hosts including humans. Nevertheless, a limited number of studies have raised the possibility that it may act as a pathogen of humans, dogs, and pigs and that some human infections may have a zoonotic origin. Data from North America revealed an association between *P. hominis* and the bovine urogenital tract, principally in bulls and rarely in cows. The importance of this observation is linked to possible interference in the accurate diagnosis of the economically important venereal pathogen *Tritrichomonas foetus*. The current study employed culture-based and molecular methods to examine the preputial cavities of four breeding bulls, raised in open pasture in southeastern Brazil, for the presence of trichomonads. Motile protozoa were isolated from three of the bulls and were definitively identified as *P. hominis* based on nucleotide sequencing of polymerase chain reaction (PCR) amplicons derived from the ribosomal RNA operon (ITS1-5.8S rDNA-ITS2) of the parasite. The potential implications of these findings for bovine and human health are discussed.

## Resumo

O parabasalídeo *Pentatrichomonas hominis* é geralmente considerado um componente simbiótico da microbiota gastrointestinal em uma ampla variedade de hospedeiros vertebrados, incluindo os humanos. No entanto, um número limitado de estudos levantou a possibilidade de que possa atuar como patógeno de humanos, cães e suínos, e que algumas infecções humanas possam ter origem zoonótica. Dados da América do Norte revelaram uma associação entre *P. hominis* e o trato urogenital bovino, principalmente, em touros e raramente em vacas. A importância dessa observação está ligada a uma possível interferência no diagnóstico preciso do patógeno venéreo de importância econômica *Tritrichomonas foetus*. O presente estudo empregou métodos baseados em cultura em combinação com técnicas moleculares, para examinar as cavidades prepuciais de quatro touros, criados em pastagem aberta no sudeste do Brasil, quanto à presença de tricomonas. Protozoários móveis foram isolados de três dos touros e foram definitivamente identificados como *P. hominis* com base no sequenciamento de nucleotídeos de amplicons geradas pela reação em cadeia da polimerase (PCR), empregando a região RNA ribossômico (ITS1-5.8S rDNA-ITS2) do parasita como alvo. Os potenciais implicações desses achados para a saúde bovina e humana são discutidas.

*Pentatrichomonas hominis* is a flagellated, anaerobic, unicellular, non-mitochondrial protozoan belonging to the *Trichomonadidae* family. It is distinguished morphologically by the presence of five anterior flagella and a single recurrent flagellum. The parasite demonstrates extensive host plasticity, having been reported as an inhabitant of the gastrointestinal tract of numerous species of vertebrate including humans, cats, cattle, dogs, goats, non-human primates, swine, owls and even reptiles, wherein it was generally considered to represent a commensal component of the gut microbiota (Li et al., 2014a, 2018, 2020; Santos et al., 2015; Mahittikorn et al., 2021). However, a limited number of studies and case reports have suggested a potential role for *P. hominis* as the causative agent of gastrointestinal disease in humans (Meloni et al., 2011) and naturally infected animals (Kim et al., 2010; Li et al., 2014b, 2020). In addition, experimental infection of pigs with *P. hominis* resulted in histological lesions associated with changes in the epithelial layer, including congestion, intestinal mucosa desquamation, severely increased numbers of inflammatory cells, vessel rupture and decreased intestinal villus length (Li et al., 2014a). In contrast, natural *P. hominis* infections in puppies presenting liquid diarrhea, weight loss, and weakness were resolved via treatment with the antiparasitic agent fenbendazole, but diarrhea persisted and the animals died, indicating that *P. hominis* was not the etiological agent of the observed gastrointestinal disturbance (Gookin et al., 2005).

An association between human gastrointestinal cancers and a significantly elevated prevalence of *P. hominis* in fecal samples in China was reported by researchers (Zhang et al., 2019), who employed a species-specific PCR assay to examine DNA isolated from feces, with no attempt made to culture the protozoa. The level of detection in cancer patients was 45%, in comparison to a level of 9% in the control population of non-cancer patients. The authors considered it probable that tumor-induced changes in the gut microflora created conditions that were favorable to colonization of the gastrointestinal tract of cancer patients with *P. hominis*, but no experimental data were provided to support this hypothesis. Moreover, they found no evidence for a pathological function for *P. hominis* in either cancer induction or progression.

In addition to gastrointestinal illness, the possible involvement of *P. hominis* in a single case of human pulmonary trichomoniasis, a rare condition normally associated with the oral parasite *Trichomonas tenax*, was reported in Thailand. *Pentatrichomonas* was detected by PCR in pleural exudates and was isolated from fecal samples, albeit no evidence of the protozoa was found in the oral cavity. Nucleotide sequencing revealed 100% sequence similarity between the amplicons generated from the pleural exudate and cultured protozoa from feces. The authors interpreted their findings as evidence for the ability of *P. hominis* to colonize body sites other than the gastrointestinal tract (Jongwutiwes et al., 2000).

Taken as a whole, the evidence to support a role for *P. hominis* as a pathogen could be viewed as controversial (Maritz et al., 2014). In contrast, there exists robust evidence demonstrating the ability of *P. hominis* to colonize the preputial cavity of bulls in North America (Hayes et al., 2003; Walker et al., 2003; Dufernez et al., 2007). In addition to confirming the ability of the protozoan to inhabit non-gastrointestinal body sites, the presence of *P. hominis* in the bovine urogenital tract, represents a problem for the accurate diagnosis of *Tritrichomonas foetus*, an economically important venereal trichomonad parasite of cattle that causes reproductive issues, including spontaneous abortion, pyometra and infertility (Bailey et al., 2021). In a subsequent study (Corbeil et al., 2008), the isolation of two non-*T. foetus* trichomonads, (identified as *P. hominis* based on the results of PCR and immunofluorescence assays) was recorded from the vaginas of breeding cows in the United States of America (USA). The same study identified the presence of *P. hominis* in the urogenital tract of breeding (non-virgin) bulls, and the authors raised the possibility that *P. hominis* may be sexually transmitted in a manner similar to *T. foetus*.

To our knowledge, no studies have been published relating the identification or isolation of *P. hominis* from the urogenital system of cattle in South America. However, colonization of the genitalia and smegma by other, non-*T. foetus*, trichomonads was reported as an infrequent phenomenon in virgin bulls reared in Argentina (Campero et al., 2003; Cobo et al., 2003). In addition, the isolation of a non-*T. foetus* trichomonad in cattle that grouped phylogenetically with the trichomonads identified in the study of Walker et al. (2003) was reported, but not discussed in detail, by Brazilian researchers (Kleina et al., 2004). The current work was conducted as part of a larger study examining the health status/presence of infectious agents, including trichomonads, in beef cattle reared in southeastern Brazil and it reports the isolation by culture and the subsequent molecular characterization of *P. hominis* from the preputial cavity of non-virgin Nelore bulls.

All procedures involving animals were conducted in strict accordance with the recommendations approved by the Ethics Commission for Research Employing Animals (CEUA/UFRRJ; Protocol nº 0130-10-2021). A total of four samples of preputial lavage were collected from individual Nelore bulls (with ages between 3.5 to 10 years old; mean age 6.3 years), raised in the municipality of Mangaratiba, Rio de Janeiro State, Brazil. The property raises beef cattle exclusively of the Nelore breed, that are farmed extensively in open pasture with mineral salts and water provided *ad libitum*. At the time of sampling in 2019, the property had a population of 909 female cattle, of which 588 were of reproductive age, and a population of eight bulls. Reproductive management was conducted through artificial insemination; however, all animals were raised in the same pasture and as such, the bulls had access to the females for natural service. All animals were certified free of brucellosis and tuberculosis and the vaccination schedule included vaccines against brucellosis, foot-and-mouth disease, rabies and clostridial diseases. In addition, all animals received routine anthelmintic treatment.

Samples were collected after cleaning the preputial region with a moist paper towel and cutting the longest and dirtiest hairs. A disposable plastic pipette coupled to a syringe with 50 mL of sterile phosphate-buffered saline (PBS; pH 7.4) was introduced into the preputial orifice, which was closed with one hand, and with the other hand, the region was massaged so that the PBS was distributed over the entire preputial mucosa. Following the massage, a 50 mL sterile Falcon^®^ tube was placed at a level below the orifice and the wash was collected by gravity. Washes were stored at ambient temperature (25ºC ± 3ºC) following collection and during transport to the laboratory (within 6 hours of collection).

Individual washes were centrifuged at 400 x *g*, for 10 min at room temperature (22°C) and the supernatant was carefully removed by aspiration to leave the pellet in approximately 1 mL of PBS. Pellets were resuspended by gently tapping the base of the tube on the benchtop and the entire material was transferred to a cell culture flask containing 6 mL of pre-warmed (37°C) Hank's^TM^ balanced salts solution (Vitrocell- Embriolife, Brazil) supplemented with 10% inactivated fetal bovine serum and streptomycin at a final concentration of 200 µg/mL, with incubation at 37°C and daily microscopic examination over seven days, commencing 24 hours post collection. Cultures identified as containing motile protozoa, via light microscopy with a 10x objective, were passaged (first subculture) using a volume of 200 µL as inoculum for a new flask containing 3 mL of pre-warmed culture solution as specified above, with incubation at 37°C and subculture performed every 72 h. Cultures showing morphologies and movement characteristic of parabasalids were subcultured over 6 passages. Aliquots (1 mL) of the third and fourth subcultures containing approximately 1 × 10^6^ trophozoites (enumerated microscopically), were collected and stored frozen (-20°C) in screw-capped microcentrifuge tubes (1.5 mL capacity) for molecular characterization.

Molecular analyses followed standardized protocols (Santos et al., 2015). Briefly, samples were defrosted, the trophozoites were sedimented by centrifugation (16,000 x *g*) and the cell pellets were washed once with 1mL of PBS (pH 7.2). Thereafter, washed cells were resuspended in 100 μL of Instagene^TM^ matrix (BIORAD) with incubation at 56°C for 30 min followed by boiling for 10 min and centrifugation (16,000 x g for 5 min) to sediment the cell debris and the chelex resin present in the Instagene^TM^ matrix. A total of 60 μL of each supernatant was transferred to 1.5 ml screw-capped microcentrifuge tubes with storage at -20 °C.

DNA extracted from the cultures suspected to contain trichomonads were examined using three distinct PCR assays. The first assay employed the primer pair “TFR1/TFR2”, and the cycling conditions reported by Felleisen (1997), in order to amplify a fragment of the ribosomal rRNA operon containing the gene encoding 5.8S ribosomal RNA and the internal transcribed spacer regions (ITS 1 and 2) of all known trichomonads. The second assay, also targeting the rRNA operon, was used to provide specific identification of *T. foetus* and employed the primer pair “TFR3/TFR4” and the cycling conditions reported by Felleisen et al. (1998). The third assay utilized the primer pair “Th3/Th5” and the cycling conditions described by Crucitti et al. (2004), being used for the specific detection of a fragment of the gene encoding the small (18S) subunit of ribosomal RNA of *P. hominis*. The positive control used in the first and second assays was DNA extracted from the *T. foetus* strain K (originally isolated from the urogenital tract of a bull, by H. Guida, Embrapa, Seropédica, Brazil). In addition, DNA extracted from a Brazilian *P. hominis* isolate recovered from a cat (Santos et al., 2015) was employed as a positive control in the first and third assays. Aliquots (5 µL) of each amplification reaction were analyzed by agarose gel electrophoresis, using 2% gels prepared in Tris-Acetic acid-EDTA (TAE) buffer. Gels were stained with ethidium bromide and visualized under ultraviolet light. The sizes of the amplification products were estimated via comparison to the positive control samples and a DNA molecular weight marker (GeneRuler, 100 bp DNA Ladder).

Post amplification analysis of amplicons was performed in two stages. Initial analysis employed a PCR-Restriction Fragment Length Polymorphism (PCR-RFLP) technique (Santos et al., 2015) to examine the amplicons of 339 bp generated using the “Th3/Th5” assay. Amplicons generated from the *P. hominis* positive control DNA and from the test samples were digested with 1 unit of the restriction enzymes *Hae*III (Promega) and *Hinf*I (Invitrogen) in individual reactions, with incubation at 37°C for 2 h. Digestion products were separated by electrophoresis on 8% polyacrylamide gels stained with ethidium bromide, visualized, and photo documented. Banding patterns were compared between samples and via comparison to a DNA molecular weight marker (GeneRuler, 50 bp DNA Ladder). The resulting images were analyzed using the free software Gel-Analyzer available at GelAnalyzer.com (2022), to accurately determine the size of individual restriction fragments.

Nucleotide sequencing of amplicons generated from the test samples using the “TFR1/TFR2” assay was performed as follows. Aliquots (10 µL) of PCR products were treated with 2 µL of ExoSap-IT (GE Healthcare) according to the manufacturer’s protocol and sequenced in both directions using the amplification primers and the BigDye Ready Reaction mix V3.1 (ABI Corp). Reaction products were analyzed using a model 3500 Automated Genetic Analyzer (ABI Corp). Sequence alignments were performed using the Sequencer^TM^ software (Version 5.6, Genecodes Corporation, USA). All sequences were entered into the BLAST search algorithm and the NCBI nucleotide database (www.ncbi.nlm.nih.gov) to determine genetic identity. A representative sequence obtained in this research was deposited in and compared with those available in the GenBank database.

The nucleotide sequence ON311004, derived from the bovine sample T1, together with 18 reference sequences representing the ITS1-5.8S rRNA-ITS2 loci of different parabasalids (including *Histomonas meleagridis* as an outgroup) were manually edited using the Sequencer^TM^ software. A phylogenetic analysis was conducted using the MEGA version11 software (Tamura et al., 2021). The evolutionary history was inferred by constructing a phylogenetic tree, tested with 1000 bootstrap replicates, using the maximum likelihood method and Kimura 2-parameter model (Kimura, 1980).

Cultures containing motile protozoa were established from three of the four preputial cavity washes. The positive cultures were denominated T1, T2 and T3. All cultures were maintained over six serial passages at which point cell viability was observed to decline. DNA was extracted from cells harvested from the third and fourth passages of each individual culture and examined by PCR. All samples produced amplicons in the assays employing the primers “TFR1/TFR2” and “Th3/Th5” but were universally negative in the assay using the primers “TFR3/TRF4”. The amplicons produced were of the same size as the bands generated by the *P. hominis* positive control DNA. These data were interpreted as suggestive of the presence of *P. hominis* in the cultures. Confirmation of colonization of the preputial cavity by *P. hominis* was obtained by two means. Firstly, amplicons of 339 bp (including the positive control) produced in the “Th3/Th5” assay were digested with the restriction enzymes resulting in the generation of fragments of 245 base pairs (bp) and 94 bp with the *Hae* III enzyme, and the production of three bands of 155 bp, 126 bp, and 53 bp with the *Hinf* I enzyme. The banding patterns observed for the T1–T3 samples were identical to those observed for the positive control amplicon. Secondly, amplicons (338 bp) derived from the test samples using the “TFR1/TFR2” assay were sequenced. High quality electropherograms were observed for all samples, indicating the presence of a single source of DNA and the sequences obtained for the three cultures were shown to be identical. The region internal to the primer binding sites (295 bp) of the sequence determined for the sample T1 was deposited in the GenBank database with the accession number (ON311004). The novel sequence showed 100% similarity (295/295 nucleotides) to sequences deposited as originating from *P. hominis* detected in human pleural exudate in Thailand (GenBank Accession number; AF156964) and canine fecal samples in France (KC623939) and the USA (AY758392).

**Figure 1:**
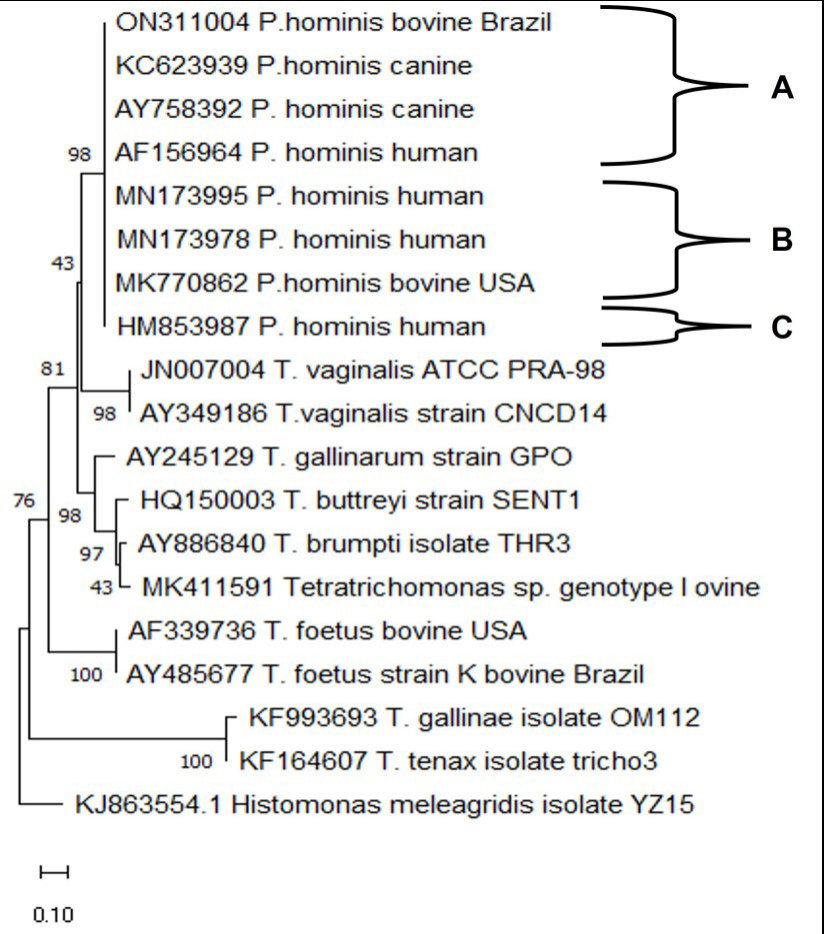
Phylogenetic tree of *P. hominis* isolates and reference parabasalid sequences of ITS1-5.8S rRNA-ITS2 loci from GenBank (375 positions in the final dataset). The evolutionary history was inferred by using the Maximum Likelihood method and Kimura 2-parameter model. The tree with the highest log likelihood (-2547.98) is shown. The percentage of trees in which the associated taxa clustered together is shown next to the branches. Initial tree(s) for the heuristic search were obtained automatically by applying Neighbor-Join and BioNJ algorithms to a matrix of pairwise distances estimated using the Maximum Composite Likelihood (MCL) approach, and then selecting the topology with superior log likelihood value. The tree is drawn to scale, with branch lengths measured in the number of substitutions per site. Scale bars represent 0.10 nucleotide substitution per site. This analysis involved 19 nucleotide sequences. Evolutionary analyses were conducted in MEGA11. **A** = Sequences with 100% nucleotide similarity with the novel Brazilian sequence ON311004; **B** = Sequences with 99.7% or 99.3% nucleotide similarity with the sequence ON311004; **C** = Sequence with 98.9% % nucleotide similarity with the sequence ON311004

A level of 99.7% sequence similarity (294/295 bp) was recorded with the sequence MK770862 (deposited by Spanish researchers) derived from a bovine isolate of *P. hominis* (ATCC 30098), recovered from cattle in the USA. Lower levels of similarity (99.3% to 98.9%) were recorded with the sequences MN173995 (293/295 bp) and MN173978 (292/295 nucleotides) detected in human feces collected from Chinese patients with gastrointestinal cancer. Details of the specific nucleotide differences observed between the sequences are provided in Table 1. Based upon the molecular analyses it was concluded that the trichomonads cultured from the preputial cavity of the bulls were *P. hominis*. Phylogenetic analysis based on the Maximum Likelihood method and using the Kimura 2-parameter evolutionary model demonstrated that the Brazilian bull-associated trichomonad sequence ON311004 clustered in the same branch as sequences derived from canine, bovine, and human isolates of *P. hominis* (Figure 1). The topology of the ML tree (Figure 1), in relation to the other species of parabasalids, was consistent with those described in previous phylogenetic studies using the ITS1-5.8S rRNA-ITS2 loci (Li et al., 2014a) or the small subunit (18S) ribosomal RNA (Mahittikorn et al., 2021).

**Table 1:** Nucleotide variance between the ITS1-5.8S rRNA-ITS2 loci of the eight *P. hominis* sequences used for phylogenetic analysis.

GenBank Accession Number				
**ON311004**	**MK770862**	**MN173995**	**MN173978**	**HM853983**
**AF156964** a				
**AY758392^a^**				
**KC623939^a^**				
17/T^b^	C	C	C	C
27/T^b^				Gap
29/T^b^				
99/T^b^		C		C
165/T^b^			A	
251/A^b^			G	

a Sequences with 100% nucleotide similarity (295/295 nt) to the sequence ON311004 derived from bovine isolate T1;

b Nucleotide position with reference to ON311004/base identified at that position

The finding of *P. hominis* colonizing the urogenital tract of non-virgin bulls in Brazil enhances the observations previously reported by North American researchers (Hayes et al., 2003; Walker et al., 2003; Dufernez et al., 2007; Corbeil et al., 2008), extending the geographical range of this phenomenon to the South American continent and adds weight to the hypothesis that this trichomonad is able to persist out with of its preferred body site. The practical implications of this finding are linked to the possible distortion of the diagnosis (based solely on culture) of *T. foetus*, and it is considered important that veterinarians and other bovine health professionals in Brazil be made aware of this association. Clearly, misdiagnosis based on morphology could be resolved using the PCR assay based on the “TFR3/TRF4” primers (Felleisen et al., 1998) to confirm the presence of *T. foetus*. Nonetheless, we recommend that the “Th3/Th5” assay should be routinely incorporated into molecular testing protocols based on the fact that the identification of *P. hominis* can be readily and cost effectively executed using the PCR-RFLP protocol employed herein (Santos et al., 2015). An additional justification for conducting complimentary molecular testing for *P. hominis* are the findings that co-infections with *T. foetus* and *P. hominis* can occur in the feline gastrointestinal tract (Santos et al., 2015), and that dual infections with *Tetratrichomonas buttreyi* and *P. hominis* were observed in 15 (1.6%) of bovine fecal samples from four farms in China, including three dairy farms and one beef farm (Li et al., 2020). Evidently, the existence of the same phenomenon in material cultured from the urogenital tract would be overlooked in the absence of specific testing for this trichomonad.

Although a matter of debate, the extensive host plasticity of *P. hominis* has been proposed as evidence for it to be considered as a zoonotic pathogen (Maritz et al, 2014; Mahittikorn et al., 2021). Thus, health professionals and farm workers having contact with the bovine urogenital tract, semen and even urine should be advised as to the potential for infection with *P. hominis* and the need to adopt appropriate biosafety procedures. The route(s) by which the protozoa became established in the preputial cavity was not determined, but as suggested by other workers (Campero et al., 2003; Hayes et al., 2003; Michi et al., 2016) transmission was most likely via contact with contaminated feces present in either the environment or in the gastrointestinal tract of cattle (including other males) which may have been mounted by the bulls. In support of this hypothesis, the presence of *P. hominis* in the bovine gastrointestinal tract has been demonstrated in Asia (Li et al., 2020; Mahittikorn et al., 2021), but to our knowledge has not been reported in the Western hemisphere.

In light of the limited number of animals examined, the findings of the current study should be considered preliminary and it is recommended that future studies on this topic should extend the range of samples tested to include the urogenital tract of cows, cattle feces present in the rearing environment and feces collected from the gastrointestinal tracts of cattle and other animals present on farms (e.g. cats and dogs) that are known hosts for *P. hominis* and that may contribute to the circulation of the protozoa within the production environment.
